# Construction and validation of a nomogram for predicting pathological upgrading/progression of gastric low-grade intraepithelial neoplasia

**DOI:** 10.3389/fmed.2026.1840288

**Published:** 2026-07-07

**Authors:** Yi-Ping Han, Hong-Li Yu, Ya-Li Wei, Yu-Bei Li, Zi-bin Tian, Xiao-Yan Yin

**Affiliations:** 1 Department of Gastroenterology, The Affiliated Hospital of Qingdao University, Qingdao, Shandong, China; 2 Department of Gastroenterology, The Fifth People's Hospital of Qingdao, Qingdao, Shandong, China; 3 Department of Gastroenterology, Qingdao West Coast New Area People's Hospital, Qingdao, Shandong, China

**Keywords:** gastric low-grade intraepithelial neoplasia, nomogram, pathological upgrading/progression, prediction model, risk factor

## Abstract

**Objective:**

To analyze the clinical, endoscopic, and pathological characteristics of gastric low-grade intraepithelial neoplasia (LGIN), identify independent risk factors for pathological upgrading/progression, and construct and internally validate a nomogram for individualized risk stratification.

**Methods:**

We retrospectively enrolled 278 patients with biopsy-confirmed gastric LGIN who underwent regular endoscopic follow-up at the Digestive Endoscopy Center of the Affiliated Hospital of Qingdao University between November 2016 and June 2023. The primary endpoint was pathological upgrading/progression, defined as either (1) true longitudinal progression from LGIN to high-grade intraepithelial neoplasia (HGIN) or gastric cancer (GC) during surveillance biopsy follow-up, or (2) histological upgrading to HGIN or GC in endoscopic submucosal dissection (ESD) specimens after an initial biopsy diagnosis of LGIN, reflecting possible biopsy underestimation. Univariable analyses were first performed, and variables with *P* < 0.05 were entered into a multivariable logistic regression model using backward stepwise selection. A nomogram was developed based on the final independent predictors. Discrimination and calibration were evaluated using the area under the receiver operating characteristic curve (AUC), bootstrap internal validation (1,000 resamples), calibration plots, and the Hosmer–Lemeshow test.

**Results:**

Pathological upgrading/progression occurred in 49 (17.63%) patients during follow-up. Multivariate analysis identified four independent risk factors for LGIN upgrading/progression: advanced age (*OR* = 1.07, 95% *CI*: 1.01–1.12, *P* = 0.018), larger lesion diameter (*OR* = 1.08, 95% *CI*: 1.03–1.15, *P* = 0.004), enlarged gastric folds (*OR* = 3.23, 95% *CI*: 1.03–10.11, *P* = 0.044), and irregular microvascular pattern (*OR* = 13.26, 95% *CI*: 1.70–103.31, *P* = 0.014). The nomogram constructed based on these factors showed good discriminative performance, with an area under the ROC curve (AUC) of 0.84 (95% *CI*: 0.78–0.89). Bootstrap internal validation (1,000 repetitions) and Hosmer–Lemeshow test (*P* = 0.333) confirmed high consistency between predicted and observed outcomes, indicating satisfactory model accuracy.

**Conclusion:**

A nomogram based on age, lesion diameter, enlarged gastric folds, and irregular microvascular pattern provides good predictive value for pathological upgrading/progression in gastric LGIN. This tool may help clinicians optimize individualized surveillance and treatment strategies. Nevertheless, because the composite endpoint includes both true progression and histological upgrading after ESD, the model should be interpreted as predicting the risk of clinically significant pathological upgrading/progression rather than pure natural-history progression alone.

## 1 Introduction

Gastric cancer (GC) is the fourth most common malignancy and the second leading cause of cancer-related mortality worldwide (1). Despite a declining incidence in recent decades, its mortality rate remains alarmingly high. Gastric carcinogenesis follows a well-defined multistep cascade: normal gastric mucosa → non-atrophic chronic gastritis → multifocal atrophic gastritis → intestinal metaplasia (IM) → low-grade intraepithelial neoplasia (LGIN) → high-grade intraepithelial neoplasia (HGIN) → invasive GC (2). As a critical pre-cancerous lesion, LGIN is characterized by epithelial cytological and architectural abnormalities without stromal invasion (3), and is classified into three subtypes of non-invasive low-grade neoplasia in the Vienna classification system (4, 5). Endoscopic resection is the standard treatment for HGIN (6), but the optimal management strategy for LGIN remains controversial due to factors such as patient compliance, cost-effectiveness, and clinical feasibility. Some lesions remain stable or regress during follow-up, whereas others are subsequently found to harbor HGIN or even carcinoma. In addition, an initial forceps biopsy diagnosis of LGIN may underestimate the true histology because of sampling limitations, and more advanced pathology may be identified in subsequent endoscopic resection specimens. Therefore, from a practical clinical perspective, two situations are relevant: true longitudinal progression during surveillance and histological upgrading identified after resection. This uncertainty often leads to either insufficient surveillance or overtreatment in clinical practice. Identifying risk factors for LGIN pathological upgrading/progression and constructing an accurate predictive model are crucial for optimizing clinical decision-making, delaying disease progression, and preventing GC. Current guidelines emphasize individualized management of gastric dysplasia according to endoscopic appearance, histology, lesion size, and background mucosal risk. However, there is still no widely accepted and clinically convenient prediction model for gastric LGIN that comprehensively integrates clinical characteristics, lesion morphology, background gastric mucosa, and magnifying endoscopy with narrow-band imaging (ME-NBI) findings. In particular, data incorporating ME-NBI microvascular features and Kyoto gastritis-related background mucosal features remain limited. Accordingly, this study retrospectively analyzed 278 patients with biopsy-confirmed gastric LGIN to identify factors associated with pathological upgrading/progression and to construct a nomogram for individualized risk assessment. In this study, the endpoint was intentionally defined as “pathological upgrading/progression” to include both true follow-up progression and histological upgrading in ESD specimens after initial biopsy, thereby reflecting the actual clinical risk faced when managing biopsy-diagnosed gastric LGIN.

## 2 Research objects and methods

### 2.1 Study population

This retrospective cohort study was approved by the Ethics Committee of the Affiliated Hospital of Qingdao University (Approval No.: QYFY WZLL 28558) and conducted in accordance with the Declaration of Helsinki.

A total of 702 patients with pathologically confirmed gastric LGIN who underwent esophagogastroduodenoscopy (EGD) at the Digestive Endoscopy Center of the Affiliated Hospital of Qingdao University from November 2016 to November 2021 were initially screened. Follow-up was continued until June 2023.

#### 2.1.1 Inclusion criteria

(1) Initial pathological diagnosis of LGIN by biopsy; (2) Regular follow-up with gastroscopy and magnifying endoscopy with narrow-band imaging (ME-NBI), with a follow-up duration >1 year; (3) Undergoing endoscopic submucosal dissection (ESD) at the end of follow-up if indicated.

#### 2.1.2 Exclusion criteria

(1) History of GC or subtotal gastrectomy; (2) Active ulcers with LGIN on biopsy; (3) Endoscopic suspicion of advanced GC but pathological confirmation of only LGIN; (4) Concomitant HGIN or GC on biopsy; (5) Diagnostic opinions differed among two or more expert physicians.

After screening, 278 patients met the criteria and were included in the analysis.

Patients received treatment according to clinical conditions, including mucosal protective agents and/or eradication therapy for *Helicobacter pylori* when indicated. Follow-up endoscopy was scheduled every 3, 6, or 12 months according to lesion characteristics and physician judgment. Biopsies were obtained from the index lesion or adjacent suspicious areas during follow-up. Baseline variables were collected from the initial endoscopic examination, and the final outcome was defined according to the latest pathology from surveillance biopsy and/or ESD specimen.

### 2.2 Outcome definition

The primary endpoint was pathological upgrading/progression, defined as: (1) True longitudinal progression: progression from LGIN to HGIN or GC confirmed by consecutive follow-up biopsies; (2) Histological upgrading: diagnosis of HGIN or GC in ESD specimens after initial biopsy diagnosis of LGIN, which may reflect biopsy underestimation. Patients who remained LGIN or regressed to normal mucosa during follow-up were classified as the non-upgrading/progression group.

### 2.3 Data collection

**Figure 1:**
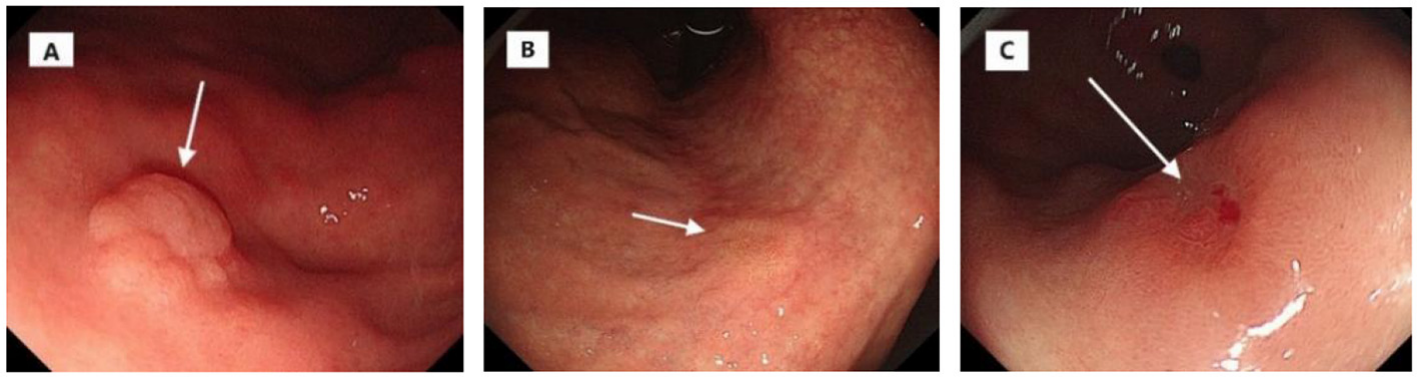
Typical images of lesion color: **(A)** white; **(B)** yellow; **(C)** red.

**Figure 2:**
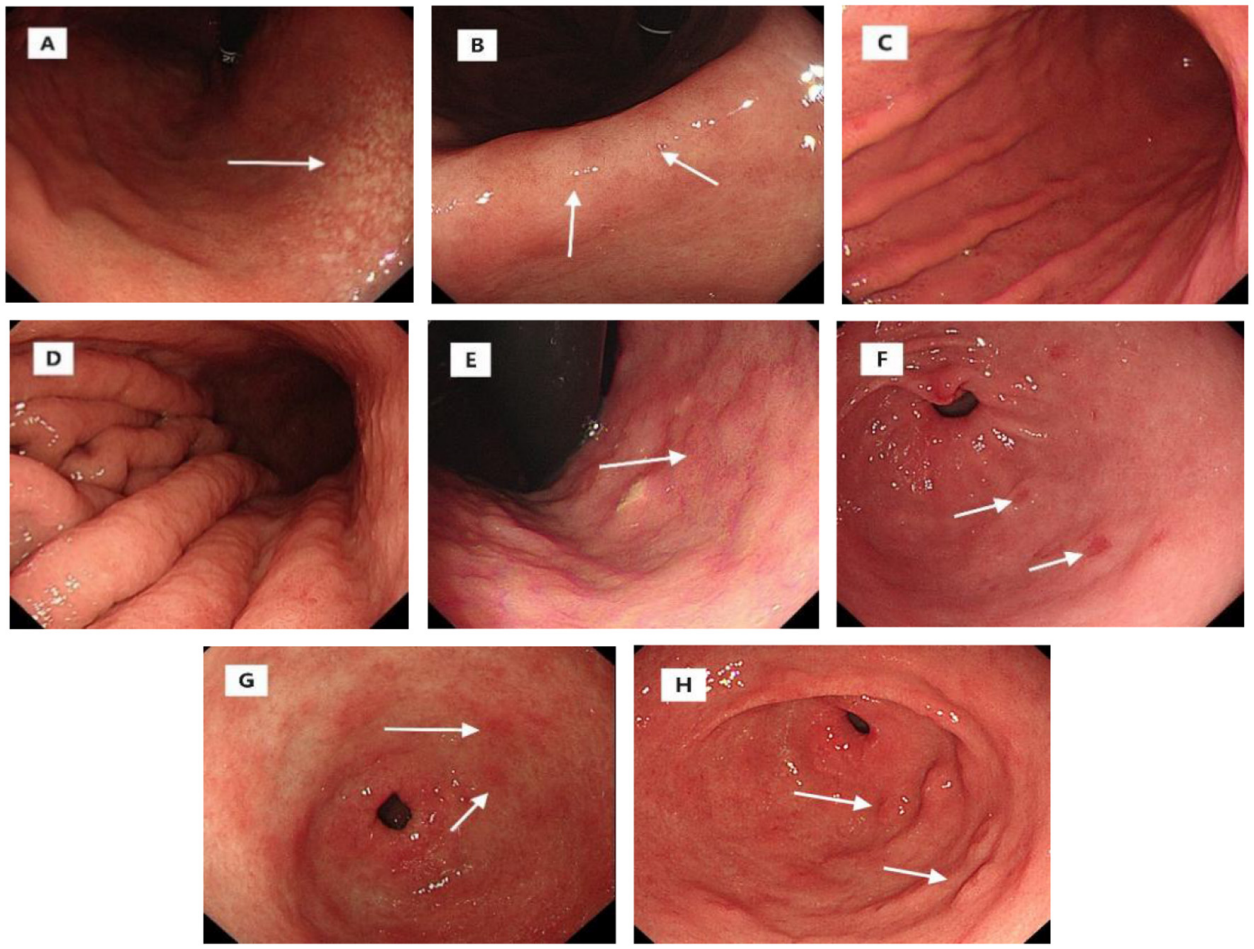
Typical endoscopic images of the gastric background mucosa: **(A)** GA: the loss of gastric glandular tissue; **(B)** IM: the replacement of native gastric epithelium with intestinal-type epithelium induced by long-term inflammation; **(C)** DR: presents as uniform, widespread erythema; **(D)** EF: thickened gastric folds with an estimated width of ≥ 5 mm under non-distended conditions that remained prominent after adequate air insufflation; **(E)** map-like redness: irregular, patchy erythematous lesions with jagged, map-like boundaries scattered on the gastric mucosa; **(F)** depression erosion: superficial loss of gastric epithelium with a visibly sunken central lesion base, surrounded by congested red mucosal margins; **(G)** spotty redness: multiple isolated, tiny punctate red foci scattered randomly over the mucosal surface, representing mild focal inflammatory hyperemia without continuous lesion fusion; **(H)** protruded: shallow epithelial erosions surrounded by raised, elevated mucosal rims.

Clinical variables included sex, age, body mass index (BMI), family history of GC, smoking history, alcohol consumption history, *H. pylori* eradication history, current *H. pylori* infection status, and treatment modality. Endoscopic variables included lesion location, size, morphology according to the Paris classification, color (Figure 1), background gastric mucosal findings based on the Kyoto classification of gastritis (Figure 2), and ME-NBI findings interpreted according to the vessel-plus-surface (VS) classification system. Background mucosal findings included gastric atrophy (GA), IM, diffuse redness, enlarged folds, map-like redness, depression erosion, spotty redness, and protruded erosion.

Operational definition of enlarged folds: enlarged folds were defined according to the Kyoto gastritis classification as thickened gastric folds with an estimated width of ≥ 5 mm under non-distended conditions that remained prominent after adequate air insufflation. In this study, a Kyoto enlarged-folds score of 1 was recorded as “yes” for the enlarged folds variable in the regression model and nomogram.

### 2.4 Endoscopic and pathological assessment

A systematic screening protocol for the stomach was used. Standardized images were obtained from the upper, middle, and lower stomach, including the gastric fundus, the greater and lesser curvature of the gastric body, the angulus, antrum, and pylorus. All procedures were performed under adequate insufflation with standardized imaging distance and exposure.

All endoscopic images were independently reviewed by three experienced endoscopists. All pathological slides were independently reviewed by three senior pathologists. Final classification was based on agreement by at least two reviewers. Cases with unresolved interpretive discrepancy after discussion were excluded from the analytic dataset. Because this approach may introduce selection bias and does not replace formal reliability assessment, this issue has been acknowledged as a study limitation.

### 2.5 Handling of missing data

Some patients had LGIN diagnosed on random biopsy without a clearly visible target lesion under endoscopy; therefore, lesion-specific endoscopic features such as size, color, morphology, and boundary were unavailable in a subset of cases. In the revised analysis, continuous lesion size was not coded as a separate “missing/NA” category. Instead, the multivariable model was fitted using patients with available data for all candidate predictors included in the final regression procedure (complete-case analysis). The number of patients included in the final multivariate model was 241 (the number of observations in the non-upgrading/progression group was 192; the number of observations in the upgrading/progression group was 49). Missingness in descriptive tables is indicated in footnotes. We acknowledge that this approach may reduce sample size and introduce bias if data were not missing completely at random; this has been added to the limitations.

### 2.6 Statistical approach

Statistical analyses were conducted using SPSS 25.0 and R 4.1.3. The Shapiro–Wilk test was used to assess the normality of continuous variables, while Levene's test was applied to evaluate homogeneity of variance. Normally distributed continuous variables were expressed as mean ± standard deviation and compared via the independent samples *t*-test; non-normally distributed variables were presented as median (interquartile range) and compared using the Mann–Whitney *U*-test. Categorical variables were summarized as frequencies and percentages, with between-group comparisons performed using the chi-square test or Fisher's exact test.

Variables yielding *P* < 0.05 in univariate analysis were included in the multivariate logistic regression model, and a backward stepwise method was adopted to screen for independent risk factors. A nomogram was subsequently established based on the identified independent predictors. The model's discriminative performance was quantified by the area under the receiver operating characteristic curve (AUC). Bootstrap resampling with 1,000 iterations was employed for internal validation and calibration curve generation, and the Hosmer–Lemeshow test was used to evaluate model calibration. *P* < 0.05 was considered statistically significant.

Justification for logistic regression: although follow-up duration varied across patients, the clinical objective of this study was to predict whether a biopsy-diagnosed gastric LGIN lesion would ultimately prove to be pathologically upgraded/progressed during the observation/resection process, rather than to model the exact timing of progression alone. Importantly, the composite endpoint included histological upgrading identified in ESD specimens, which does not fit a pure time-to-event framework in the same way as natural-history progression observed under surveillance. Therefore, logistic regression was selected to model the occurrence of clinically relevant pathological upgrading/progression over the study period. This methodological choice and its limitations have been explicitly acknowledged in the Discussion.

## 3 Results

### 3.1 Clinical outcome of biopsy in patients with low-grade intraepithelial neoplasia

**Figure 3:**
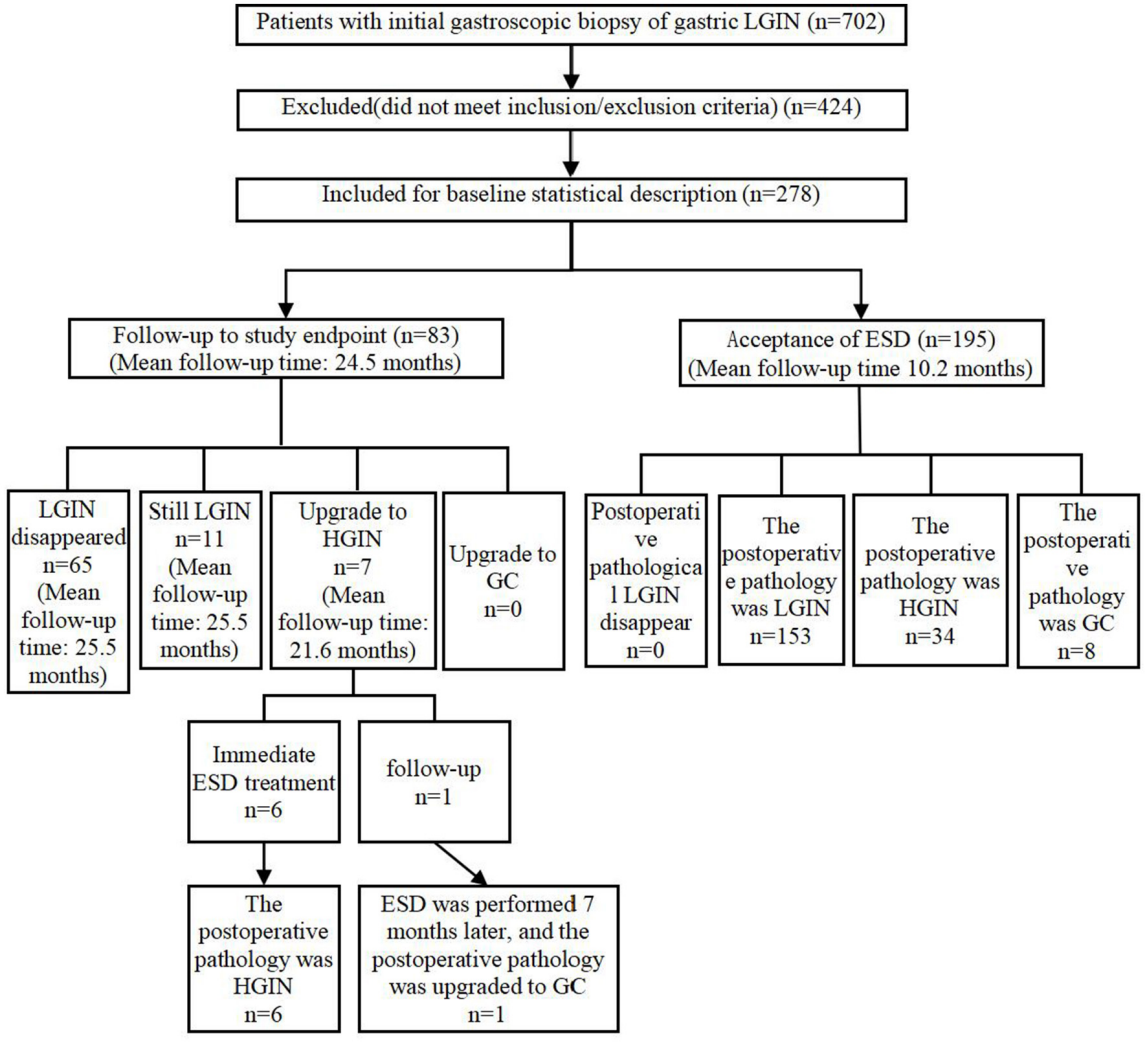
Clinical outcomes of patients with low-grade intraepithelial neoplasia by biopsy.

A total of 702 patients with gastric LGIN were initially screened. Overall, 424 patients failed to meet the inclusion and exclusion criteria, among whom 15 were excluded due to interpretive disagreement among experts. The remaining 278 patients were included for baseline statistical description. The mean age was 61.1 years, and 175 (62.9%) were male. During follow-up, 49 patients (17.63%) progressed pathologically to HGIN or GC, and 229 patients maintained stable or improved conditions (Figure 3).

### 3.2 Analysis of risk factors for LGIN upgrading/progression

Univariate analysis revealed significant differences in clinical characteristics between the upgrading/progression and non-upgrading/progression group: age (*P* = 0.004), sex (*P* = 0.020), *H. pylori* eradication history (*P* = 0.026), and current *H. pylori* infection (*P* = 0.007).

Endoscopic characteristics associated with upgrading/progression included lesion size (*P* < 0.001), lesion location (*P* = 0.015), and irregular microvascular/microsurface pattern (*P* < 0.001). Background mucosal characteristics such as atrophy (*P* = 0.017), IM (*P* = 0.030), enlarged folds (*P* < 0.001), and Kyoto gastritis score (*P* < 0.001) were also significantly associated with progression.

No significant differences were found in family history of GC, smoking/alcohol history, BMI, treatment modality, lesion morphology/color, diffuse redness, erosions, map-like redness, mottled congestion, or lesion margin characteristics between the two groups (all *P* > 0.05; Table 1).

**Table 1:** Univariate analysis of risk for upgrading/progression of gastric low-grade intraepithelial neoplasia.

Parameters	Non-upgrading/ progression group (*n* = 229)	Upgrading/progression group (*n* = 49)	*P*	*X* ^2^	95% CI
Age, years, mean ± SD	60.448 ± 0.322	64.187 ± 0.831	0.004		−4.543 to 0.686
Sex, man, *n* (%)	137 (59.8)	38 (77.6)	0.020	5.438	
Family history of gastric cancer, yes, *n* (%)	62 (27.1)	13 (26.5)	0.938	0.006	
Smoking history, yes, *n* (%)	61 (26.6)	17 (34.7)	0.255	1.298	
Drinking history, yes, *n* (%)	49 (21.4)	13 (26.5)	0.433	0.614	
BMI, kg/m^2^, mean ± SD	24.062 ± 0.724	23.552 ± 0.854	0.260		−0.383 to 1.396
Treatment, *n* (%)			0.419	2.829	
Follow-up	107 (46.7)	21 (42.9)			
Mucosal protective	46 (20.1)	15 (30.6)			
*H. pylori* eradication	37 (16.2)	7 (14.3)			
Stomach protection combined with bactericidal	39 (17.0)	6 (12.2)			
History of eradication of *H. pylori, n* (%)			0.026	7.273	
Uninfected	98 (42.8)	15 (30.6)			
Unsterilized	55 (24.0)	21 (42.9)			
After sterilizing	76 (33.2)	13 (26.5)	0.039^#^		
Current *H. pylori* infection, positive, *n* (%)	55 (24.0)	21 (42.9)	0.007	7.212	
Size, mm^*^, mean ± SD	10 ± 7	15 ± 8	< 0.001		−8.258 to −2.926
Location, *n* (%)			0.015	8.019	
Upper	4 (1.8)	5 (10.2)			
Middle	68 (29.7)	16 (32.7)			
Lower	157 (68.6)	28 (57.1)			
Morphology, *n* (%)^*^			0.177	5.877	
Is	4 (2.1)	1 (2.0)			
IIa	103 (53.7)	18 (36.7)			
IIb	1 (0.5)				
IIc	65 (33.9)	25 (51.0)			
IIa + IIc	19 (9.9)	5 (10.2)			
Color, *n* (%)^*^			0.423	1.682	
Red	93 (48.4)	29 (59.2)			
Yellowish	79 (41.1)	16 (32.7)			
White	20 (10.4)	4 (8.2)			
GA, *n* (%)			0.017	9.727	
No	2 (0.9)	1 (2.0)			
C1–C2	97 (42.4)	13 (26.5)			
C3–O1	118 (51.5)	27 (55.1)			
O2–O3	12 (5.2)	8 (16.3)			
IM, *n* (%)			0.030	6.436	
IM0	2 (0.9)	1 (2.0)			
IM1	45 (19.7)	3 (6.1)			
IM2	182 (79.5)	45 (91.8)			
DR, yes, *n* (%)	164 (71.6)	40 (81.6)	0.150	2.073	
EF, yes, *n* (%)	76 (33.2)	33 (67.3)	< 0.001	19.760	
Depression erosion, yes, *n* (%)	95 (41.5)	17 (34.7)	0.379	0.774	
Protrude erosion, yes, *n* (%)	85 (37.1)	11 (22.5)	0.050	3.842	
Map-like redness, yes, *n* (%)	78 (34.1)	18 (36.7)	0.721	0.128	
Spotty redness, yes, *n* (%)	137 (59.8)	30 (61.2)	0.856	0.033	
Kyoto gastritis score, *M* (IQR)	4 (2)	5 (2)	< 0.001		
Boundary, *n* (%)^*^			1.000		
Clear	189 (98.4)	48 (98.0)			
Unclear	3 (1.6)	1 (2.0)			
Microsurface and microvascular, irregular, *n* (%)	158 (69.0)	48 (98.0)	< 0.001	17.643	

^#^In the history of H. pylori eradication, the difference between the two groups after and without sterilization was statistically significant (P = 0.039 < 0.05).

^*^Because some patients with no obvious lesions under endoscopy had LGIN according to random biopsy, partial data on size, color, morphology, and boundaries were missing.

The number of observations in the non-upgrading/progression group was 192.

The number of observations in the upgrading/progression group was 49.

Multivariate logistic regression analysis identified four independent risk factors for LGIN pathological upgrading/progression: (1) Advanced age (*OR* = 1.07, 95% *CI*: 1.01–1.12, *P* = 0.018); (2) Larger lesion diameter (*OR* = 1.08, 95% *CI*: 1.03–1.15, *P* = 0.004); (3) Enlarged gastric folds (*OR* = 3.23, 95% *CI*: 1.03–10.11, *P* = 0.044); (4) Irregular microvascular pattern (*OR* = 13.26, 95% *CI*: 1.70–103.31, *P* = 0.014; Table 2). Sensitivity analysis using Firth penalized logistic regression yielded consistent results, with all four factors remaining statistically significant, confirming the stability of the model.

**Table 2:** Multivariate analysis of the risk of gastric low-grade intraepithelial neoplasia upgrading/progression^&^.

Variable	Regression coefficient (*B*)	SE	*P*	OR	95% CI
Age, per year	0.063	0.026	0.018	1.065	1.011–1.121
Sex					
Female				1	
Male	0.816	0.425	0.055	2.262	0.983–5.209
History of eradication of *H. pylori*					
Uninfected				1	
Unsterilized	0.780	0.460	0.090	2.182	0.885–5.381
After sterilizing	0.230	0.490	0.639	1.258	0.482–3.285
Current *H. pylori* infection					
Negative				1	
Positive	0.682	0.407	0.093	1.979	0.891–4.391
Lesion size, per mm	0.080	0.028	0.004	1.084	1.026–1.145
Location					
Upper	0.820	0.842	0.330	2.271	0.436–11.839
Middle	−0.459	0.436	0.292	0.632	0.269–1.484
Lower				1	
GA					
No				1	
Mild (C1.C2)	−1.529	1.785	0.392	0.217	0.007–7.175
Moderate (C3.O1)	−1.772	1.787	0.321	0.170	0.005–5.646
Severe (O2.O3)	−0.927	1.992	0.642	0.396	0.008–19.624
IM					
IM2				1	
IM1	−0.824	0.912	0.366	0.439	0.073–2.621
EF					
Absent				1	
Present	1.172	0.582	0.044	3.229	1.031–10.108
Kyoto gastritis score	0.094	0.251	0.707	1.099	0.672–1.798
Microsurface and microvascular					
Regular				1	
Irregular	2.585	1.047	0.014	13.259	1.702–103.308

^&^Variables with an odds ratio (OR) of 1 were defined as reference categories.

Categorical variables were numerically coded as follows: sex (1 = male, 2 = female), H. pylori eradication history (0 = uninfected, 1 = unsterilized, 2 = after sterilizing), current H. pylori infection status (0 = negative, 1 = positive), lesion location (1 = upper stomach, 2 = middle stomach, 3 = lower stomach), gastric mucosal atrophy (0 = absent, 1 = mild [C1/C2], 2 = moderate [C3/O1], 3 = severe [O2/O3]), IM (1 = IM1, 2 = IM2), EF (0 = absent, 1 = present), and microsurface and microvascular patterns (1 = regular, 2 = irregular).

### 3.3 Construction and validation of nomograms for predicting the pathological upgrading/progression of LGIN

**Figure 4:**
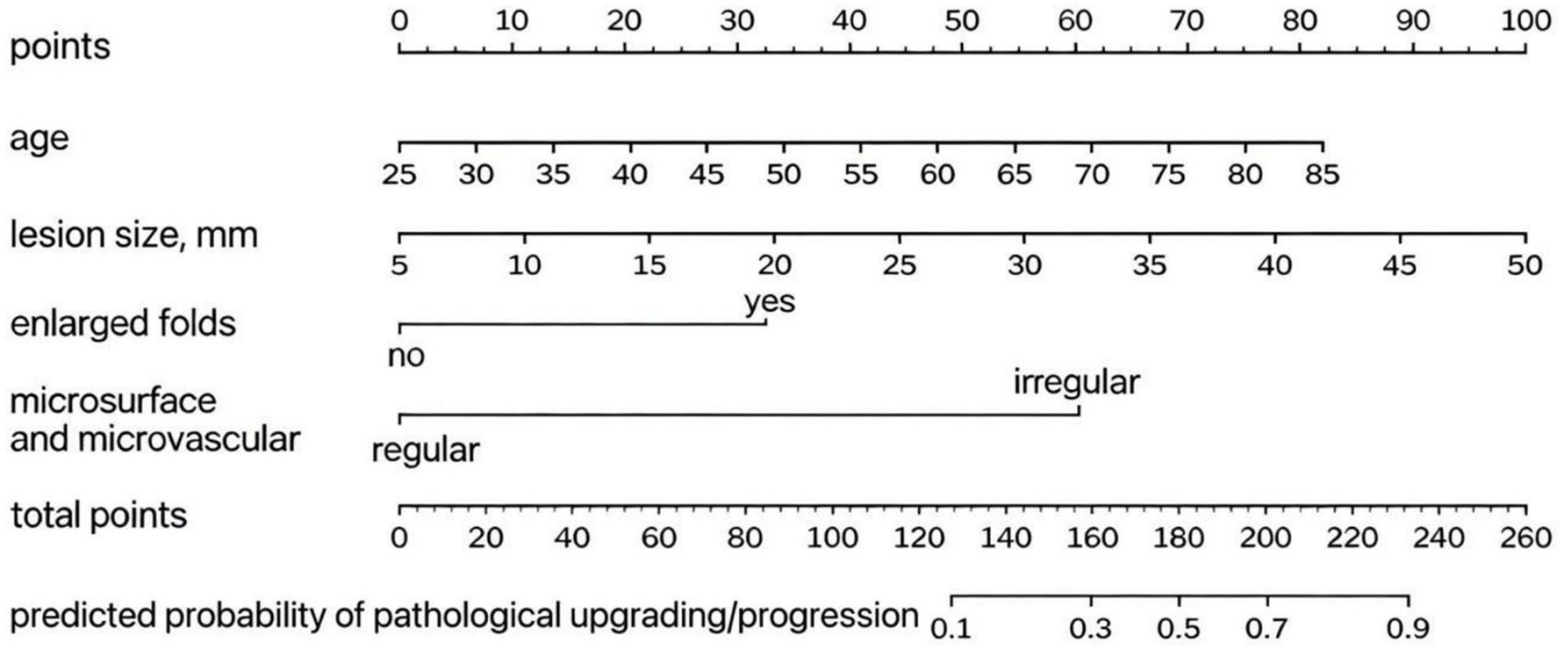
Model for predicting the pathological upgrading/progression of gastric low-grade intraepithelial neoplasia-nomogram.

**Figure 5:**
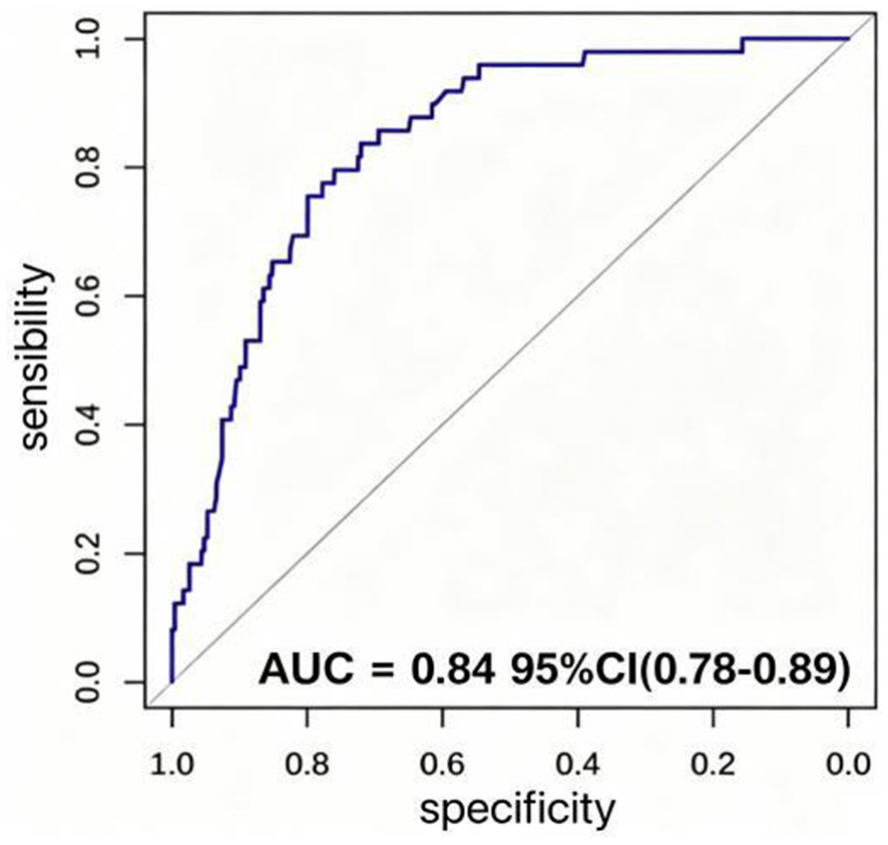
ROC curve.

**Figure 6:**
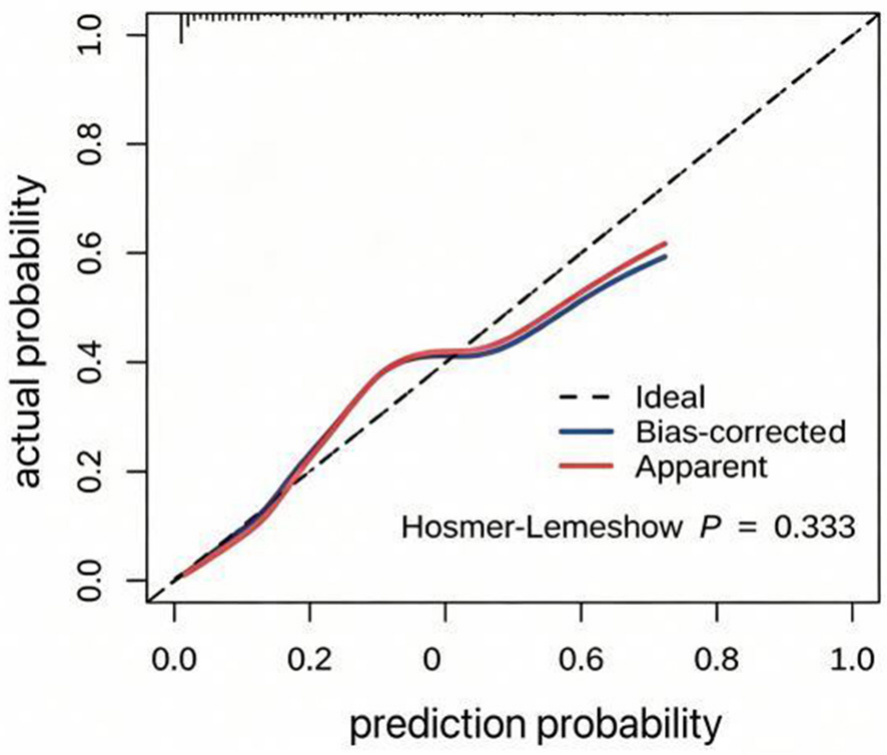
Calibration curve.

Based on the four independent risk factors, a nomogram prediction model was constructed (Figure 4). The nomogram showed good discriminative performance, with an AUC of 0.84 (95% *CI*: 0.78–0.89; Figure 5). Bootstrap internal validation (1,000 repetitions) generated a calibration curve (Figure 6) demonstrating high consistency between predicted and observed upgrading/progression risks. The Hosmer–Lemeshow test result (*P* = 0.333) further confirmed the model's satisfactory accuracy.

## 4 Discussion

Gastric LGIN is a critical pre-cancerous lesion in the gastric carcinogenic cascade, and its pathological upgrading/progression to HGIN or GC poses a significant threat to patient prognosis. In this study, 17.63% of LGIN patients experienced pathological upgrading/progression during follow-up, highlighting the need for accurate risk stratification.

A major strength of the present study is the explicit use of “pathological upgrading/progression” as the endpoint. In clinical practice, a biopsy diagnosis of LGIN may represent either a truly low-risk lesion that remains stable under surveillance or an under-sampled lesion whose more advanced histology is only revealed in an ESD specimen. From the perspective of patient management, both scenarios are clinically important because both indicate that the initial biopsy diagnosis underestimated the lesion's ultimate pathological significance. Nevertheless, these two phenomena are not identical biologically. True longitudinal progression reflects temporal evolution, whereas histological upgrading after ESD may reflect baseline sampling error. Therefore, our model should be interpreted as predicting the overall risk that a biopsy-diagnosed LGIN lesion will ultimately be upgraded/progressed pathologically, rather than pure natural-history progression alone.

We identified four independent risk factors for LGIN upgrading/progression: advanced age, larger lesion diameter, enlarged gastric folds, and irregular microvascular pattern. Advanced age and male sex were associated with increased upgrading/progression risk, consistent with previous studies (7, 8). The age-related increase in GC risk may be due to cumulative mucosal damage and genomic instability (9), while gender disparities may be attributed to environmental, occupational, and physiological differences (10).

*H. pylori* infection is a key etiological factor for gastric pre-cancerous lesions (2). Although univariate analysis showed that current *H. pylori* infection was associated with upgrading/progression, it was not an independent predictor in multivariate analysis. This may be because *H. pylori* eradication can interrupt the pre-cancerous cascade (2, 6), and active inflammation may mask LGIN features, leading to under-diagnosis (11, 12). Thus, *H. pylori* eradication should be prioritized in LGIN patients, and follow-up strategies should be individualized based on background mucosal status.

Lesion location analysis revealed that LGIN lesions were most commonly located in the gastric lower third (66.5%), but lesions in the upper gastric region had a significantly higher progression rate (55.6%) than those in the middle (19.0%) and lower (5.1%) regions, consistent with Ruan et al. (13). Larger lesion diameter was an independent risk factor, which may be related to the accumulation of genetic mutations in larger lesions.

ME-NBI is a valuable tool for evaluating gastric mucosal microstructures. In our study, irregular microvascular pattern was a strong independent risk factor (*OR* = 13.26), with 98.0% of progression patients presenting this feature. This is consistent with previous reports (14, 15), confirming that ME-NBI can effectively stratify LGIN upgrading/progression risk, Although the confidence interval was wide due to sparse data, Firth regression confirmed its independent predictive value. Enlarged gastric folds, a novel independent risk factor identified in this study, may reflect severe chronic inflammation or pre-cancerous mucosal changes, warranting close surveillance.

This study has several limitations. First, it was a single-center retrospective study with a limited sample size and only 49 endpoint events, which increased the risk of model overfitting. To address this concern, we reduced the final model to a parsimonious set of predictors, performed bootstrap internal validation. External validation in independent cohorts remains necessary before clinical implementation. Second, because the endpoint combined true longitudinal progression and histological upgrading after ESD, the model does not isolate the natural history of untreated LGIN. Third, logistic regression was used instead of a time-to-event model. This choice was based on the composite clinical endpoint and the practical aim of predicting whether a lesion would eventually be pathologically upgraded/progressed; however, variation in follow-up duration and possible censoring may still affect risk estimation. Future studies with larger samples should consider separate analyses for true progression and ESD-based upgrading, ideally using time-to-event methods where appropriate. Fourth, some lesion-specific endoscopic variables were missing in patients diagnosed by random biopsy without visible focal lesions. In the revised analysis, complete-case modeling was used rather than treating missing continuous values as a separate category, but this may still introduce bias. Fifth, final image and pathology interpretation was based on expert consensus, and cases with unresolved discrepancy were excluded. This may have introduced selection bias and potentially inflated apparent model performance. Therefore, formal inter-observer agreement statistics and prospective standardized assessment should be incorporated in future studies.

In conclusion, we developed a nomogram based on age, lesion size, enlarged folds, and irregular microvascular pattern to predict pathological upgrading/progression in gastric LGIN. This model may assist risk stratification and help guide individualized surveillance and endoscopic treatment decisions, but further external validation is required.

## Data Availability

The raw data supporting the conclusions of this article will be made available by the authors, without undue reservation.
